# Bis(di-2-pyridyl­methane­diol-κ^3^ *N*,*O*,*N*′)nickel(II) dibenzoate

**DOI:** 10.1107/S160053681003669X

**Published:** 2010-09-18

**Authors:** Jin Hoon Kim, Du-Hyun Kim, Pan-Gi Kim, Cheal Kim, Youngmee Kim

**Affiliations:** a Department of Fine Chemistry, and Eco-Product and Materials Education Center, Seoul National University of Science and Technology, Seoul 139-743, Republic of Korea; b Department of Forest Resources Development, Korea Forest Research Institute, Suwon 441-350, Republic of Korea; c Department of Forest & Environment Resources, Kyungpook National University, Sangju,742-711, Republic of Korea; d Department of Chemistry and Nano Science, Ewha Womans University, Seoul 120-750, Republic of Korea

## Abstract

The title compound, [Ni(C_11_H_10_N_2_O_2_)_2_](C_7_H_5_O_2_)_2_, consists of an Ni^II^ ion coordinated by two tridentate chelating (2-py)_2_C(OH)_2_ ligands (py is pyrid­yl) and two benzoate anions. The Ni^II^ ion is located on a twofold rotation axis, and its geometry is distorted octa­hedral. The *gem*-diol ligand (2-py)_2_C(OH)_2_ adopts an η^1^:η^1^:η^1^ coordination mode. There are O—H⋯O hydrogen bonds between the *gem*-diol ligands and benzoate anions.

## Related literature

For examples of inter­actions between transition metal ions and biologically active mol­ecules, see: Efthymiou *et al.* (2006 ▶); Daniele *et al.* (2008 ▶); Parkin (2004 ▶); Tshuva & Lippard (2004 ▶). For related structures of Cu(II) and Zn(II) benzoates, see: Lee *et al.* (2008 ▶); Yu *et al.* (2008 ▶); Park *et al.* (2008 ▶); Shin *et al.* (2009 ▶); Yu *et al.* (2010 ▶). For the di-2-pyridyl­ketone [(py)_2_CO] ligand, see: Papaefstathiou & Perlepes (2002 ▶); Stoumpos *et al.* (2009 ▶). For related structures, see: Wang *et al.* (1986 ▶); Li *et al.* (2005 ▶); Yu *et al.* (2009*a* ▶,*b* ▶).
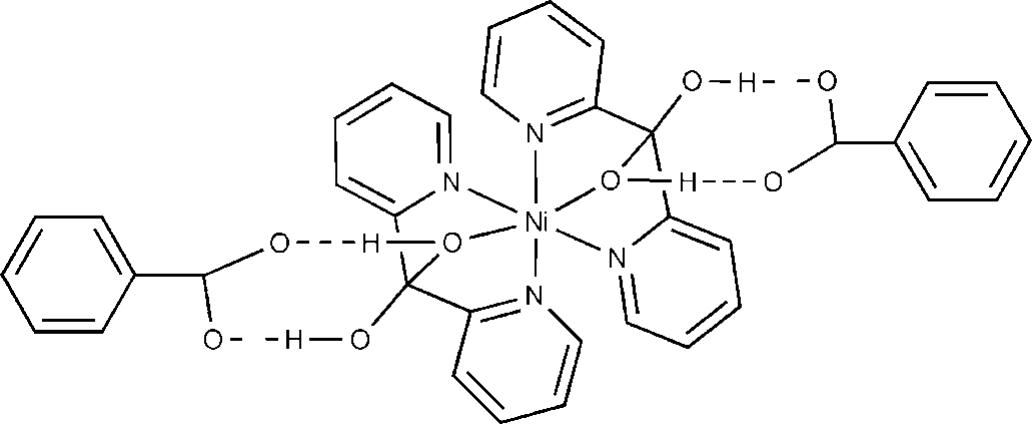


## Experimental

### 

#### Crystal data

[Ni(C_11_H_10_N_2_O_2_)_2_](C_7_H_5_O_2_)_2_
*M* *_r_* = 705.35
Monoclinic, 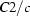

*a* = 24.065 (8) Å
*b* = 8.681 (3) Å
*c* = 17.718 (6) Å
β = 123.526 (5)°
*V* = 3085.7 (17) Å^3^
*Z* = 4
Mo *K*α radiation
μ = 0.69 mm^−1^
*T* = 173 K
0.08 × 0.05 × 0.05 mm

#### Data collection

Bruker SMART CCD diffractometer
Absorption correction: multi-scan (*SADABS*; Bruker, 1997 ▶) *T*_min_ = 0.959, *T*_max_ = 0.966
8329 measured reflections
3031 independent reflections
1818 reflections with *I* > 2σ(*I*)
*R*_int_ = 0.095

#### Refinement

*R*[*F* ^2^ > 2σ(*F* ^2^)] = 0.057
*wR*(*F* ^2^) = 0.109
*S* = 1.01
3031 reflections
222 parameters
H-atom parameters constrained
Δρ_max_ = 0.38 e Å^−3^
Δρ_min_ = −0.34 e Å^−3^

### 

Data collection: *SMART* (Bruker, 1997 ▶); cell refinement: *SAINT* (Bruker, 1997 ▶); data reduction: *SAINT*; program(s) used to solve structure: *SHELXS97* (Sheldrick, 2008 ▶); program(s) used to refine structure: *SHELXL97* (Sheldrick, 2008 ▶); molecular graphics: *ORTEPIII* (Burnett & Johnson, 1996 ▶) and *ORTEP-3 for Windows* (Farrugia, 1997 ▶); software used to prepare material for publication: *SHELXL97*.

## Supplementary Material

Crystal structure: contains datablocks I, global. DOI: 10.1107/S160053681003669X/dn2601sup1.cif

Structure factors: contains datablocks I. DOI: 10.1107/S160053681003669X/dn2601Isup2.hkl

Additional supplementary materials:  crystallographic information; 3D view; checkCIF report

## Figures and Tables

**Table 1:** Hydrogen-bond geometry (Å, °)

*D*—H⋯*A*	*D*—H	H⋯*A*	*D*⋯*A*	*D*—H⋯*A*
O1—H1*O*⋯O21	0.84	1.70	2.537 (3)	171
O2—H2*O*⋯O22	0.84	1.79	2.615 (4)	167

**Fig. 1.**
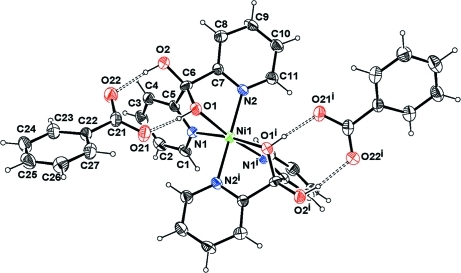
Structure of the title complex with labeling scheme. Displacement ellipsoids are shown at the 50% probability level. H atoms are represented as small sphere of arbitrary radii and hydrogen bonds are shown as dashed line [Symmetry code: (i) -x+1, y, -z+3/2]

## supplementary crystallographic information

### Comment

The interaction of transition metal ions with biologically active molecules such as amino acids, proteins, sugars, fulvic acids and humic acids is of great importance in the biological systems (Daniele, *et al.*, 2008; Parkin, 2004; Tshuva and Lippard, 2004). As models to examine the interaction, the study on the interaction of the transition metal ions with various acids such as benzoic acid has been intensively examined. Our group have also reported a variety of structures of copper(II) and zinc(II) benzoates with quinoxaline, 6-methylquinoline, 3-methylquinoline, *trans*-1-(2-pyridyl)-2-(4-pyridyl)ethylene, and di-2-pyridyl ketone (Lee, *et al.*, 2008; Yu, *et al.*, 2008; Park, *et al.*, 2008; Shin, *et al.*,2009; Yu, *et al.*, 2009*a*,*b*; Yu, *et al.*,2010).

Di-2-pyridyl ketone ((py)_2_CO) has been employed to form structurally interesting new complexes with 3 d-metal ions (Stoumpos, *et al.*, 2009). While the neutral ligands (py)_2_C(OH)_2_ and (py)_2_C(OR)(OH) coordinate to the metal centres as N,N',O chelates (Papaefstathiou and Perlepes, 2002), water and alcohols (ROH) have been shown to add to the carbonyl group forming the ligands (2-py)_2_C(OH)_2 _[the *gem*-diol form of (2-py)_2_CO] and (2-py)_2_C(OR)(OH) [the hemiacetal form of (2-py)_2_CO], respectively (Efthymiou *et al.*, 2006). The Ni(II) complexes of the neutral ligand, (py)_2_C(OH)_2_ have been characterized (Wang, *et al.*, 1986; Li, *et al.*, 2005; Yu, *et al.*, 2009*a*,*b*), but no structure with a benzoate ion as the counter-ion has been reported. We report here another structure of Ni^II^ benzoate containing a neutral ligand (2-py)_2_C(OH)_2_.

The Ni^II^ atom is coordinated by two tridentate chelating (2-py)_2_C(OH)_2_ ligand to form a distorted octahedral geometry. The Ni^II^ ion is located on a two fold axis. The *gem*-diol ligand (2-py)_2_C(OH)_2 _adopts the coordination mode η^1^:η^1^:η^1^ (Fig.1). There are hydrogen bonds between the *gem*-diol hydrogen atoms and benzoate oxygen atoms.

### Experimental

36.4 mg (0.125 mmol) of Ni(NO_3_)_2_^.^6H_2_O and 35.5 mg (0.25 mmol) of C_6_H_5_COONH_4_ were dissolved in 4 ml water and carefully layered by 4 ml solution of a mixture of acetone, methanol and ethanol (1/1/1) of di-2-pyridyl ketone ligand (46.1 mg, 0.25 mmol). Suitable crystals of the title compoundfor X-ray analysis were obtained in a month.

### Refinement

H atoms were placed in calculated positions and treated as riding on their parent atoms  with C—H distances of 0.93 Å (phenyl) and 0.84 Å (hydroxyl) and U_iso_(H) = 1.2U_eq_(C) or 1.5U_eq_(O).

### Crystal data

**Table:**

[Ni(C_11_H_10_N_2_O_2_)_2_](C_7_H_5_O_2_)_2_	*F*(000) = 1464
*M**_r_* = 705.35	*D*_x_ = 1.518 Mg m^−^^3^
Monoclinic, *C*2/*c*	Mo *K*α radiation, λ = 0.71073 Å
Hall symbol: -C 2yc	Cell parameters from 526 reflections
*a* = 24.065 (8) Å	θ = 2.6–18.8°
*b* = 8.681 (3) Å	µ = 0.69 mm^−^^1^
*c* = 17.718 (6) Å	*T* = 173 K
β = 123.526 (5)°	Block, colourless
*V* = 3085.7 (17) Å^3^	0.08 × 0.05 × 0.05 mm
*Z* = 4	

### Data collection

**Table:**

Bruker SMART CCD diffractometer	3031 independent reflections
Radiation source: fine-focus sealed tube	1818 reflections with *I* > 2σ(*I*)
graphite	*R*_int_ = 0.095
φ and ω scans	θ_max_ = 26.0°, θ_min_ = 2.0°
Absorption correction: multi-scan (*SADABS*; Bruker, 1997)	*h* = −29→28
*T*_min_ = 0.959, *T*_max_ = 0.966	*k* = −10→9
8329 measured reflections	*l* = −20→21

### Refinement

**Table:**

Refinement on *F*^2^	Primary atom site location: structure-invariant direct methods
Least-squares matrix: full	Secondary atom site location: difference Fourier map
*R*[*F*^2^ > 2σ(*F*^2^)] = 0.057	Hydrogen site location: inferred from neighbouring sites
*wR*(*F*^2^) = 0.109	H-atom parameters constrained
*S* = 1.01	*w* = 1/[σ^2^(*F*_o_^2^) + (0.*P*)^2^] where *P* = (*F*_o_^2^ + 2*F*_c_^2^)/3
3031 reflections	(Δ/σ)_max_ < 0.001
222 parameters	Δρ_max_ = 0.38 e Å^−^^3^
0 restraints	Δρ_min_ = −0.33 e Å^−^^3^

### Special details

**Table:**

Geometry. All esds (except the esd in the dihedral angle between two l.s. planes) are estimated using the full covariance matrix. The cell esds are taken into account individually in the estimation of esds in distances, angles and torsion angles; correlations between esds in cell parameters are only used when they are defined by crystal symmetry. An approximate (isotropic) treatment of cell esds is used for estimating esds involving l.s. planes.
Refinement. Refinement of *F*^2^ against ALL reflections. The weighted *R*-factor *wR* and goodness of fit *S* are based on *F*^2^, conventional *R*-factors *R* are based on *F*, with *F* set to zero for negative *F*^2^. The threshold expression of *F*^2^ > σ(*F*^2^) is used only for calculating *R*-factors(gt) *etc*. and is not relevant to the choice of reflections for refinement. *R*-factors based on *F*^2^ are statistically about twice as large as those based on *F*, and *R*- factors based on ALL data will be even larger.

### Fractional atomic coordinates and isotropic or equivalent isotropic displacement parameters (Å^2^)

**Table:**

	*x*	*y*	*z*	*U*_iso_*/*U*_eq_	
Ni1	0.5000	0.54475 (8)	0.7500	0.0218 (2)	
O1	0.42807 (11)	0.6969 (3)	0.74041 (15)	0.0272 (6)	
H1O	0.4294	0.7180	0.7877	0.041*	
O2	0.31141 (11)	0.6882 (3)	0.65747 (15)	0.0311 (7)	
H2O	0.3095	0.6916	0.7034	0.047*	
N1	0.43794 (14)	0.3935 (4)	0.75897 (18)	0.0240 (8)	
N2	0.43061 (13)	0.5602 (3)	0.61309 (17)	0.0224 (7)	
C1	0.45080 (19)	0.2545 (5)	0.7972 (2)	0.0295 (10)	
H1	0.4945	0.2135	0.8250	0.035*	
C2	0.4030 (2)	0.1682 (5)	0.7979 (2)	0.0340 (10)	
H2	0.4136	0.0696	0.8257	0.041*	
C3	0.33914 (19)	0.2273 (5)	0.7573 (2)	0.0336 (10)	
H3	0.3053	0.1696	0.7564	0.040*	
C4	0.32564 (18)	0.3714 (5)	0.7182 (2)	0.0282 (10)	
H4	0.2823	0.4147	0.6904	0.034*	
C5	0.37576 (16)	0.4519 (5)	0.7200 (2)	0.0231 (9)	
C6	0.36850 (16)	0.6119 (4)	0.6785 (2)	0.0244 (9)	
C7	0.36976 (17)	0.5939 (4)	0.5939 (2)	0.0215 (9)	
C8	0.31530 (18)	0.6127 (4)	0.5068 (2)	0.0278 (9)	
H8	0.2725	0.6345	0.4949	0.033*	
C9	0.32512 (19)	0.5986 (4)	0.4372 (2)	0.0306 (10)	
H9	0.2886	0.6117	0.3763	0.037*	
C10	0.38729 (18)	0.5658 (5)	0.4555 (2)	0.0326 (10)	
H10	0.3944	0.5566	0.4081	0.039*	
C11	0.43897 (17)	0.5467 (5)	0.5446 (2)	0.0286 (9)	
H11	0.4821	0.5230	0.5580	0.034*	
O21	0.42695 (12)	0.7889 (3)	0.87550 (16)	0.0364 (7)	
O22	0.31909 (12)	0.7353 (3)	0.80877 (16)	0.0409 (8)	
C21	0.3730 (2)	0.7889 (5)	0.8723 (3)	0.0306 (10)	
C22	0.37566 (18)	0.8581 (5)	0.9521 (2)	0.0272 (9)	
C23	0.33218 (19)	0.8072 (5)	0.9755 (3)	0.0329 (10)	
H23	0.3011	0.7277	0.9411	0.039*	
C24	0.3337 (2)	0.8705 (5)	1.0476 (3)	0.0382 (11)	
H24	0.3041	0.8339	1.0632	0.046*	
C25	0.3776 (2)	0.9862 (5)	1.0971 (3)	0.0396 (11)	
H25	0.3776	1.0312	1.1459	0.047*	
C26	0.42184 (18)	1.0377 (5)	1.0767 (3)	0.0356 (10)	
H26	0.4527	1.1170	1.1119	0.043*	
C27	0.42110 (18)	0.9730 (5)	1.0042 (3)	0.0342 (10)	
H27	0.4519	1.0077	0.9903	0.041*	

### Atomic displacement parameters (Å^2^)

**Table:**

	*U*^11^	*U*^22^	*U*^33^	*U*^12^	*U*^13^	*U*^23^
Ni1	0.0214 (4)	0.0260 (4)	0.0186 (4)	0.000	0.0115 (3)	0.000
O1	0.0272 (14)	0.0340 (17)	0.0230 (13)	−0.0008 (13)	0.0155 (12)	−0.0029 (13)
O2	0.0259 (15)	0.0429 (19)	0.0280 (14)	0.0089 (13)	0.0170 (12)	0.0053 (13)
N1	0.0235 (18)	0.029 (2)	0.0212 (16)	0.0028 (15)	0.0131 (14)	0.0027 (15)
N2	0.0260 (17)	0.0239 (19)	0.0186 (15)	0.0032 (15)	0.0131 (13)	0.0025 (15)
C1	0.034 (2)	0.032 (3)	0.021 (2)	0.003 (2)	0.0150 (18)	0.006 (2)
C2	0.045 (3)	0.031 (3)	0.028 (2)	−0.001 (2)	0.021 (2)	0.005 (2)
C3	0.039 (3)	0.038 (3)	0.027 (2)	−0.016 (2)	0.020 (2)	−0.005 (2)
C4	0.027 (2)	0.035 (3)	0.023 (2)	−0.003 (2)	0.0144 (18)	−0.007 (2)
C5	0.024 (2)	0.029 (2)	0.0179 (18)	−0.003 (2)	0.0125 (16)	−0.0023 (19)
C6	0.0156 (19)	0.031 (2)	0.022 (2)	−0.0003 (17)	0.0067 (16)	−0.0016 (19)
C7	0.023 (2)	0.020 (2)	0.023 (2)	−0.0006 (16)	0.0138 (17)	0.0000 (16)
C8	0.026 (2)	0.027 (2)	0.027 (2)	−0.0004 (18)	0.0124 (18)	−0.0006 (19)
C9	0.037 (2)	0.033 (3)	0.0168 (19)	0.0050 (19)	0.0116 (18)	0.0022 (18)
C10	0.040 (2)	0.037 (3)	0.024 (2)	0.007 (2)	0.0197 (19)	0.002 (2)
C11	0.030 (2)	0.031 (2)	0.026 (2)	0.005 (2)	0.0168 (17)	0.001 (2)
O21	0.0333 (16)	0.044 (2)	0.0411 (16)	−0.0030 (14)	0.0264 (13)	−0.0072 (14)
O22	0.0316 (17)	0.062 (2)	0.0295 (15)	−0.0064 (15)	0.0172 (13)	−0.0119 (15)
C21	0.034 (2)	0.032 (3)	0.036 (2)	0.004 (2)	0.025 (2)	0.004 (2)
C22	0.028 (2)	0.031 (3)	0.027 (2)	0.0065 (19)	0.0184 (18)	0.003 (2)
C23	0.037 (2)	0.032 (3)	0.036 (2)	0.002 (2)	0.024 (2)	0.002 (2)
C24	0.046 (3)	0.046 (3)	0.032 (2)	0.002 (2)	0.028 (2)	0.002 (2)
C25	0.053 (3)	0.039 (3)	0.033 (2)	0.014 (2)	0.027 (2)	0.003 (2)
C26	0.039 (2)	0.030 (3)	0.039 (2)	0.002 (2)	0.022 (2)	−0.004 (2)
C27	0.036 (2)	0.034 (3)	0.042 (2)	0.004 (2)	0.028 (2)	0.004 (2)

### Geometric parameters (Å, °)

**Table:**

Ni1—N2^i^	2.053 (3)	C6—C7	1.525 (5)
Ni1—N2	2.053 (3)	C7—C8	1.374 (5)
Ni1—N1	2.060 (3)	C8—C9	1.383 (5)
Ni1—N1^i^	2.060 (3)	C8—H8	0.9500
Ni1—O1^i^	2.109 (3)	C9—C10	1.374 (5)
Ni1—O1	2.109 (2)	C9—H9	0.9500
O1—C6	1.437 (4)	C10—C11	1.377 (5)
O1—H1O	0.8408	C10—H10	0.9500
O2—C6	1.378 (4)	C11—H11	0.9500
O2—H2O	0.8405	O21—C21	1.267 (4)
N1—C1	1.334 (4)	O22—C21	1.247 (4)
N1—C5	1.353 (4)	C21—C22	1.506 (5)
N2—C7	1.340 (4)	C22—C27	1.388 (5)
N2—C11	1.340 (4)	C22—C23	1.393 (5)
C1—C2	1.379 (5)	C23—C24	1.373 (5)
C1—H1	0.9500	C23—H23	0.9500
C2—C3	1.385 (5)	C24—C25	1.368 (5)
C2—H2	0.9500	C24—H24	0.9500
C3—C4	1.379 (5)	C25—C26	1.373 (5)
C3—H3	0.9500	C25—H25	0.9500
C4—C5	1.379 (5)	C26—C27	1.393 (5)
C4—H4	0.9500	C26—H26	0.9500
C5—C6	1.535 (5)	C27—H27	0.9500
			
N2^i^—Ni1—N2	172.49 (18)	O2—C6—C7	110.0 (3)
N2^i^—Ni1—N1	95.84 (11)	O1—C6—C7	104.5 (3)
N2—Ni1—N1	88.95 (11)	O2—C6—C5	113.4 (3)
N2^i^—Ni1—N1^i^	88.95 (11)	O1—C6—C5	107.3 (3)
N2—Ni1—N1^i^	95.84 (11)	C7—C6—C5	108.6 (3)
N1—Ni1—N1^i^	100.79 (17)	N2—C7—C8	122.8 (3)
N2^i^—Ni1—O1^i^	76.59 (10)	N2—C7—C6	112.6 (3)
N2—Ni1—O1^i^	98.62 (10)	C8—C7—C6	124.5 (3)
N1—Ni1—O1^i^	172.42 (10)	C7—C8—C9	117.6 (4)
N1^i^—Ni1—O1^i^	78.91 (11)	C7—C8—H8	121.2
N2^i^—Ni1—O1	98.62 (10)	C9—C8—H8	121.2
N2—Ni1—O1	76.59 (10)	C10—C9—C8	120.5 (3)
N1—Ni1—O1	78.91 (11)	C10—C9—H9	119.7
N1^i^—Ni1—O1	172.42 (10)	C8—C9—H9	119.7
O1^i^—Ni1—O1	102.40 (14)	C9—C10—C11	118.1 (3)
C6—O1—Ni1	99.7 (2)	C9—C10—H10	120.9
C6—O1—H1O	110.2	C11—C10—H10	120.9
Ni1—O1—H1O	118.6	N2—C11—C10	122.3 (3)
C6—O2—H2O	109.5	N2—C11—H11	118.8
C1—N1—C5	118.7 (3)	C10—C11—H11	118.8
C1—N1—Ni1	129.9 (2)	O22—C21—O21	124.8 (4)
C5—N1—Ni1	111.5 (3)	O22—C21—C22	118.6 (3)
C7—N2—C11	118.6 (3)	O21—C21—C22	116.6 (3)
C7—N2—Ni1	112.1 (2)	C27—C22—C23	118.4 (4)
C11—N2—Ni1	129.3 (2)	C27—C22—C21	121.3 (3)
N1—C1—C2	122.4 (4)	C23—C22—C21	120.2 (4)
N1—C1—H1	118.8	C24—C23—C22	120.7 (4)
C2—C1—H1	118.8	C24—C23—H23	119.6
C1—C2—C3	119.0 (4)	C22—C23—H23	119.6
C1—C2—H2	120.5	C25—C24—C23	120.2 (4)
C3—C2—H2	120.5	C25—C24—H24	119.9
C4—C3—C2	118.9 (4)	C23—C24—H24	119.9
C4—C3—H3	120.6	C24—C25—C26	120.6 (4)
C2—C3—H3	120.6	C24—C25—H25	119.7
C3—C4—C5	119.2 (4)	C26—C25—H25	119.7
C3—C4—H4	120.4	C25—C26—C27	119.6 (4)
C5—C4—H4	120.4	C25—C26—H26	120.2
N1—C5—C4	121.9 (4)	C27—C26—H26	120.2
N1—C5—C6	112.7 (3)	C22—C27—C26	120.4 (4)
C4—C5—C6	125.5 (3)	C22—C27—H27	119.8
O2—C6—O1	112.6 (3)	C26—C27—H27	119.8

Symmetry codes: (i) −*x*+1, *y*, −*z*+3/2.

### Hydrogen-bond geometry (Å, °)

**Table:**

*D*—H···*A*	*D*—H	H···*A*	*D*···*A*	*D*—H···*A*
O1—H1O···O21	0.84	1.70	2.537 (3)	171
O2—H2O···O22	0.84	1.79	2.615 (4)	167
